# Genome Sequence of *Streptomyces* sp. H-KF8, a Marine Actinobacterium Isolated from a Northern Chilean Patagonian Fjord

**DOI:** 10.1128/genomeA.01645-16

**Published:** 2017-02-09

**Authors:** Agustina Undabarrena, Juan Antonio Ugalde, Eduardo Castro-Nallar, Michael Seeger, Beatriz Cámara

**Affiliations:** a Laboratorio de Microbiología Molecular y Biotecnología Ambiental, Departamento de Química & Centro de Biotecnología Daniel Alkalay Lowitt Universidad Técnica Federico Santa María, Valparaíso, Chile; b Centro de Genética y Genómica, Facultad de Medicina, Clínica Alemana Universidad del Desarrollo, Santiago, Chile; c Centro de Bioinformática y Biología Integrativa, Facultad de Ciencias Biológicas, Universidad Andrés Bello, Santiago, Chile

## Abstract

*Streptomyces* sp. H-KF8 is a fjord-derived marine actinobacterium capable of producing antimicrobial activity. *Streptomyces* sp. H-KF8 was isolated from sediments of the Comau fjord, located in the northern Chilean Patagonia. Here, we report the 7.7-Mb genome assembly, which represents the first genome of a Chilean marine actinobacterium.

## GENOME ANNOUNCEMENT

Members of the genus *Streptomyces* are widely recognized for producing a plethora of bioactive secondary metabolites with antimicrobial, antifungal, and antitumoral properties (1). As marine environments are markedly different from terrestrial ones, it has been proposed that marine *Streptomyces* spp. may produce different types of bioactive compounds in comparison to their terrestrial counterparts (2).

The extensive coast of Chile is especially attractive for exploring marine actinobacterial communities. The remote Comau fjord, located in the northern Chilean Patagonia is a marine-protected area suitable for bioprospection due to its unique geologic nature. There are scarce reports within this area that involve the characterization of microbial communities from water samples (3), underwater microbial mats (4), and terrestrial hot spring mats (5, 6). Previously, we aimed to isolate actinobacteria from marine sediments obtained from various coastal locations at different depths and evaluate their antimicrobial potential (7). Among these, the marine actinobacterium *Streptomyces* sp. H-KF8, isolated from 15-m-deep marine sediments obtained from Punta Llonco, Comau fjord, was selected for whole-genome sequencing. A prominent activity against *Staphylococcus aureus*, *Listeria monocytogenes*, and *Escherichia coli* was previously determined (7), and therefore, *Streptomyces* sp. H-KF8 is an interesting candidate to explore for drug discovery.

DNA extraction was performed with the Wizard Genomic DNA extraction kit (Promega). Next-generation sequencing data were provided by Macrogen and generated by Illumina HiSeq2000 (paired-end library of 2 × 100 bp) and PacBio (library construction of 5-kb average size and one SMRT cell with a P5-C3 chemistry) technologies. The whole genome was *de novo* assembled using Canu version 1.1 (8) and consisted of 11 scaffolds represented in one linear chromosome with a total of 7,684,888 bp, a GC content of 72.1%, and coverage of 500× (*N*_50_, 4,115,122 bp; mean read length, 698,626 bp). The genome was annotated using the NCBI Prokaryotic Genome Annotation Pipeline (PGAP) version 3.1 (https://www.ncbi.nlm.nih.gov/genome/annotation_prok), leading to a total of 6,574 genes assigned as follows: 6,486 coding sequences, 67 tRNAs, 18 rRNAs, and three ncRNAs.

An antiSMASH version 3.0 search (9) led to the identification of 26 biosynthetic gene clusters (BGCs) for secondary metabolites, including two polyketide synthases (PKSs), two nonribosomal peptides synthetases (NRPSs), and four hybrid PKS-NRPSs, which may be involved in the antimicrobial activity previously observed (7). Only 23% of the clusters have 100% similarity with other known BGCs. Further genomic analysis of these secondary metabolism routes may provide insights into the production of interesting candidates for natural product discovery.

To our knowledge, this is the first report of whole-genome sequencing of a marine *Streptomyces* strain in Chile, which may assist future comparative genomics studies. Next-generation sequencing techniques play a fundamental role in elucidating the biotechnological potential of environmental isolates, revealing their cryptic genetic features.

### Accession number(s).

The *Streptomyces* sp. H-KF8 genome sequence was deposited in GenBank under the accession number LWAB00000000.

## References

[1] Bérdy J 2012 Thoughts and facts about antibiotics: where we are now and where we are heading. J Antibiot 65:441–441. doi:10.1038/ja.2012.54. 22922479

[2] Manivasagan P, Venkatesan J, Sivakumar K, Kim SK 2013 Marine actinobacterial metabolites: current status and future perspectives. Microbiol Res 168:311–332. doi:10.1016/j.micres.2013.02.002. 23480961

[3] Elizondo-Patrone C, Hernández K, Yannicelli B, Olsen LM, Molina V 2015 The response of nitrifying microbial assemblages to ammonium (NH_4_^+^) enrichment from salmon farm activities in a northern Chilean fjord. Estuar Coast Shelf Sci 166:131–142. doi:10.1016/j.ecss.2015.03.021.

[4] Ugalde JA, Gallardo MJ, Belmar C, Muñoz P, Ruiz-Tagle N, Ferrada-Fuentes S, Espinoza C, Allen EE, Gallardo VA 2013 Microbial life in a fjord: metagenomic analysis of a microbial mat in Chilean Patagonia. PLoS One 8:e71952. doi:10.1371/journal.pone.0071952. 24015199 PMC3756073

[5] Mackenzie R, Pedrós-Alió C, Díez B 2013 Bacterial composition of microbial mats in hot springs in Northern Patagonia: variations with seasons and temperature. Extremophiles 17:123–136. doi:10.1007/s00792-012-0499-z. 23208511

[6] Estrella Alcamán M, Fernandez C, Delgado A, Bergman B, Díez B 2015 The cyanobacterium *Mastigocladus* fulfills the nitrogen demand of a terrestrial hot spring microbial mat. ISME J 9:2290–2303. doi:10.1038/ismej.2015.63. 26230049 PMC4579480

[7] Undabarrena A, Beltrametti F, Claverías FP, González M, Moore ER, Seeger M, Cámara B 2016 Exploring the diversity and antimicrobial potential of marine *Actinobacteria* from the Comau fjord in northern Patagonia, Chile. Front Microbiol 7:1135. doi:10.3389/fmicb.2016.01135. 27486455 PMC4949237

[8] Berlin K, Koren S, Chin CS, Drake JP, Landolin JM, Phillippy AM 2015 Assembling large genomes with single-molecule sequencing and locality-sensitive hashing. Nat Biotechnol 33:623–630. doi:10.1038/nbt.3238. 26006009

[9] Weber T, Blin K, Duddela S, Krug D, Kim HU, Bruccoleri R, Lee SY, Fischbach MA, Müller R, Wohlleben W, Breitling R, Takano E, Medema MH 2015 antiSMASH 3.0—a comprehensive resource for the genome mining of biosynthetic gene clusters. Nucleic Acids Res 43:W237–W243. doi:10.1093/nar/gkv437. 25948579 PMC4489286

